# Phosphorylated peptide of G protein-coupled receptor induces dimerization in activated arrestin

**DOI:** 10.1038/s41598-020-67944-0

**Published:** 2020-07-02

**Authors:** Andreas M. Stadler, Joachim Granzin, Anneliese Cousin, Renu Batra-Safferling

**Affiliations:** 1 Jülich Centre for Neutron Science (JCNS-1), Institute of Complex Systems (ICS-1), Forschungszentrum Jülich, 52425 Jülich, Germany; 2 Institute of Physical Chemistry, RWTH Aachen University, Landoltweg 2, 52074 Aachen, Germany; 3 Institute of Biological Information Processing, IBI-7: Structural Biochemistry, Forschungszentrum Jülich, 52425 Jülich, Germany

**Keywords:** Biochemistry, Biophysics, Structural biology

## Abstract

Termination of the G-protein-coupled receptor signaling involves phosphorylation of its C-terminus and subsequent binding of the regulatory protein arrestin. In the visual system, arrestin-1 preferentially binds to photoactivated and phosphorylated rhodopsin and inactivates phototransduction. Here, we have investigated binding of a synthetic phosphopeptide of bovine rhodopsin (residues 323–348) to the active variants of visual arrestin-1: splice variant p44, and the mutant R175E. Unlike the wild type arrestin-1, both these arrestins are monomeric in solution. Solution structure analysis using small angle X-ray scattering supported by size exclusion chromatography results reveal dimerization in both the arrestins in the presence of phosphopeptide. Our results are the first report, to our knowledge, on receptor-induced oligomerization in arrestin, suggesting possible roles for the cellular function of arrestin oligomers. Given high structural homology and the similarities in their activation mechanism, these results are expected to have implications for all arrestin isoforms.

## Introduction

G protein-coupled receptors (GPCRs) constitute the largest class of membrane receptors that mediate the majority of cellular responses upon binding to a diverse category of external stimuli such as light, peptides, hormones, neurotransmitters, chemokines and growth factors. Activation of GPCRs mostly initiates G-protein dependent signaling where the GPCR interacts with a heterotrimeric G protein, triggering the downstream signaling activities^1^. Termination of the GPCR-mediated signaling involves a two-step arrestin-mediated desensitization, where activated receptor is first phosphorylated by a G protein-coupled receptor kinase (GRK) followed by high affinity binding of arrestin that sterically blocks the interaction between receptor and G-protein, leading to the signal termination.

**Figure 1:**
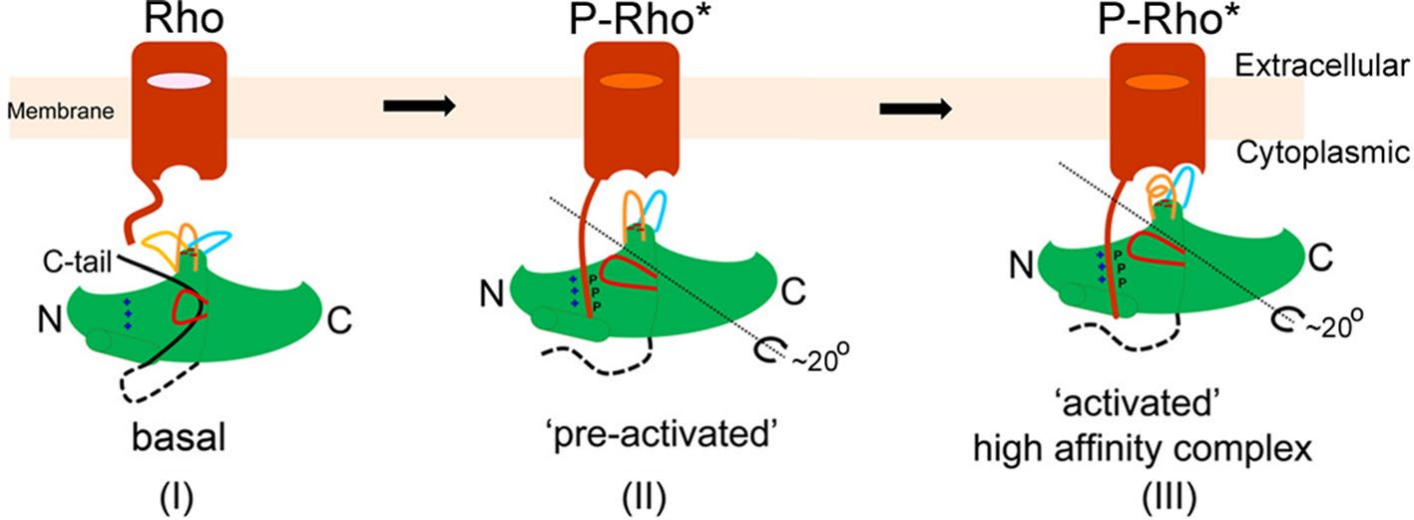
Role of receptor C-terminal phosphopeptide in arrestin activation. In the cartoon model, three conformations of arr-1 are depicted namely basal, pre-activated and activated. Basal state of arr-1 is the inactive conformation, primarily stabilized by a close network of interactions between residues in the polar core, N-terminus and the C-tail. Activation of rhodopsin (P-Rho*) allows binding of phosphorylated C-terminus to the N-domain of arr-1, disrupting the polar core network and releasing the C-tail. The resulting ‘pre-activated’ arr-1 state is typically characterized by structural differences in the central crest loop conformations and a ~ 20° rotation between the N- and C-domains. Consequently, opening of the loop 139 (middle loop) and C-loop leads to accommodation of ICL2 helix of rhodopsin, forming the high-affinity activated state of arr-1. Associated to fully-activated state of arr-1 is the α-helical conformation of the finger loop at the interface.

Arrestins are a small family of four homologous proteins, consisting two members each of visual and non-visual (beta) arrestins. In addition to desensitization of GPCRs, members of the arrestin family are also involved in receptor internalization and in stimulating G-protein independent signaling pathways. Crystal structures of all four subtypes of arrestins^2–6^ as well as of a few arrestin-GPCR complexes^7–9^ are known where high similarity is observed in the structures as well as in the binding mechanism of arrestins with their respective GPCRs^10^. The structural basis of arrestin activation is depicted in the cartoon model (Fig. 1). In the basal state, arrestin is in the inactive conformation, primarily stabilized by a close network of interactions between residues in the polar core, N-terminus and the C-tail. Activation of the receptor allows binding of its phosphorylated C-terminus to the N-domain of arrestin, disrupting the polar core network and releasing the C-tail of arrestin. The bound phosphorylated C-terminus of the receptor forms an extended intermolecular β sheet with the N-terminal β-strands of arrestin^8,9^. The resulting ‘active’ arrestin conformation is typically characterized by structural differences in the central crest loop conformations and a rotation (up to  ~ 20º) between the N- and C-domains. Consequently, opening of the loop 139 (middle loop) and C-loop leads to accommodation of ICL2 helix of the receptor, forming the high-affinity activated state of arrestin, with the α-helical finger loop at the interface.

Splice variants of arrestin have been reported previously for rod arrestin and β-arrestin^11–15^. These are generated through alternative mRNA splicing event, yielding truncated isoforms of the respective arrestin. p44 is the splice variant of visual arrestin (arr-1), which differs from arrestin only in the C-terminus where it lacks the last 35 amino acids that are replaced by an alanine residue^13^. Localized permanently in the rod outer segments (ROS)^13^, p44 has been shown to exist in pre-activated state^14^; and shows a higher affinity for light-activated phosphorylated rhodopsin (P-Rh*) than arr-1^16^. In contrast to arr-1 that binds specifically to P-Rh*, p44 has selectivity for both photolyzed and phosphorylated rhodopsin^12,13,16^.

Interaction between visual arrestin (arr-1) and synthetic phosphopeptides derived from the C-terminus of GPCR rhodopsin show a tight binding (K_D_ < 250 μM)^17,18^. In the presented paper, we have investigated binding of a synthetic phosphopeptide to active variants of arr-1: polar core mutant R175E and splice variant p44 using small angle X-ray scattering. In contrast to the dimeric wild type arr-1, both these arrestins are monomeric in solution^19^. Our results reveal dimerization in both the active arrestin variants in the presence of phosphopeptide. Though self-association in arrestin family members is known^20^, the work presented here demonstrates receptor-induced oligomerization in pre-activated arrestins.

## Results

**Figure 2:**
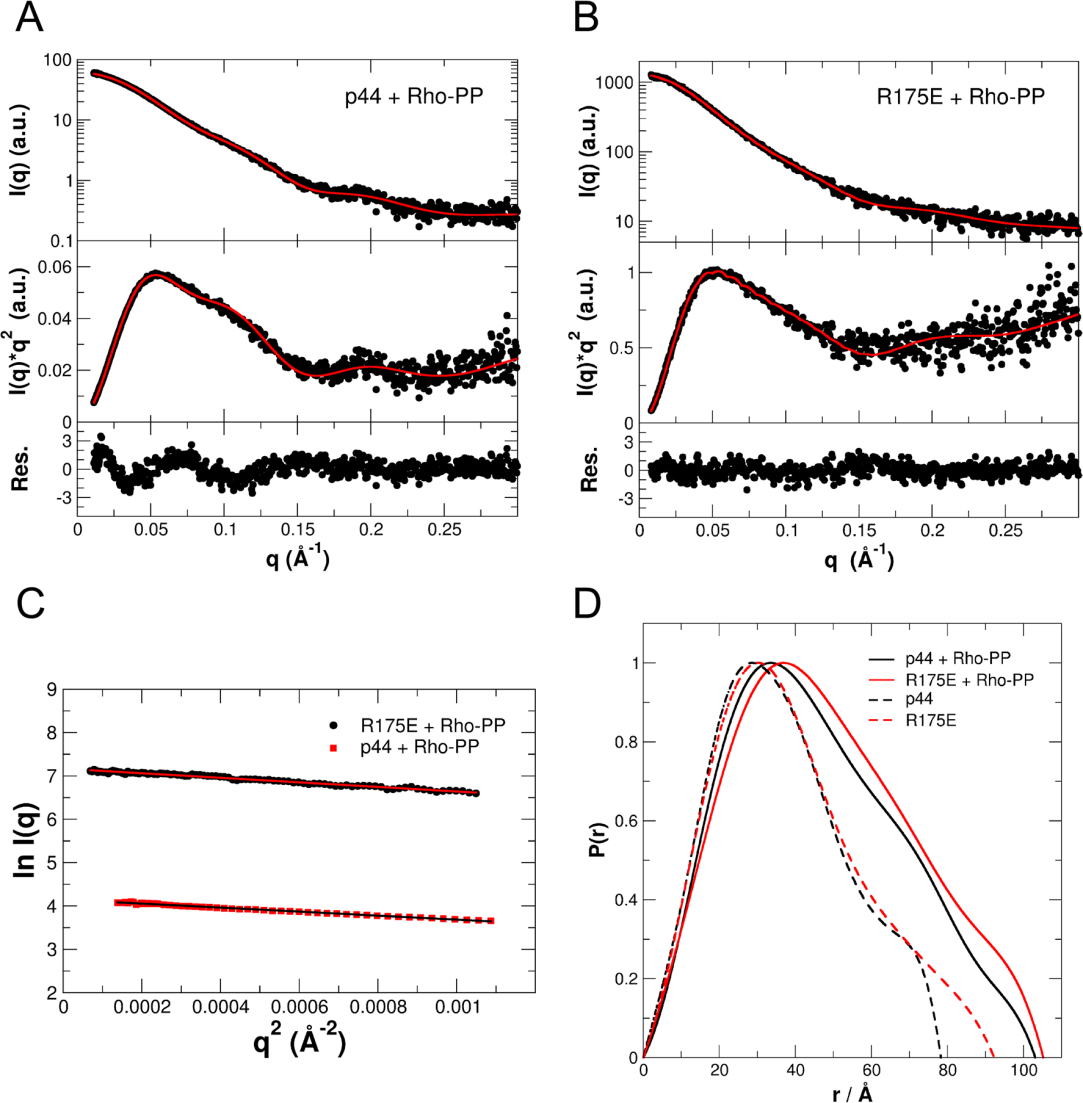
Small-angle scattering data of peptide bound arrestins: p44 (**A**) and R175E (**B**). Experimental data and theoretical scattering curves (solid lines) using the program CRYSOL of the respective crystal structures (PDB ID for p44: 3UGU) and EOM (PDB ID for R175E: 4ZRG) including the flexible ends. The upper panels visualize the measured data on a logarithmic scale, while Kratky plots are shown in the middle panels. The lower panels display the residuals to illustrate the goodness of the structure-based fits to the experimental data. Peptide binding results in dimerization of p44 and R175E and the crystal structure based dimeric arrangements are shown in Fig. 3 (C–C dimer, as in PDB 4J2Q). (**C**) Guinier plots of both the data sets shown in panels A and B. (**D**) Pair distribution functions P(r) of p44 and R175E arrestin in the presence of phosphopeptide. p44 + Rho-PP (black line); R175E + Rho-PP (red line). Pair distribution function P(r) of monomeric p44 (black) and R175E (red) in the absence of phosphopeptide are shown as dashed lines.

In the current study, we aimed to perform SAXS measurements to investigate the effect of rhodopsin phosphopeptide (Rho-PP) binding on the active states of arr-1, namely, p44 and R175E mutant in solution; and to determine the low resolution structures of respective complexes. For the purpose, p44 and R175E were measured using SAXS in the absence and presence of the phosphopeptide. Experimental SAXS data of p44 + Rho-PP and R175E + Rho-PP are shown in Fig. 2 (Fig. 2A,B). Guinier plots of both data sets are shown in Fig. 2C. In parallel, p44 and R175E without the peptide were measured as the respective references. Experimental SAXS data of p44 and R175E without peptide are shown in the supporting information (Supplementary Fig S1). Both the proteins are monomeric in solution in the absence of phosphopeptide, these results have been reported previously^19^. Pair distribution functions P(r) have been calculated from the measured SAXS data. P(r) functions of p44 and R175E with bound phosphopeptide, and of the monomeric p44 and R175E in the absence of phosphopeptide are given in Fig. 2D.

Primary physical parameters such as Guinier radii R_G_, maximal dimension of the protein D_max_, and molecular mass estimates based on Porod volume, Bayesian interference and obtained from ab initio modelling are summarized in Table 1 for p44 and R175E in the absence and presence of phosphopeptide. Theoretical molecular masses calculated from the amino acid sequences of the respective monomer and dimers are also given (Table 1). The comparison of measured molecular mass estimates with the corresponding theoretical values demonstrates that in the absence of phosphopeptide both proteins are in their monomeric state. In contrast, the interaction with the phosphopeptide induces dimerization in both the arrestins as demonstrated by an increase of particle size as well by the increase of R_G_ and D_max_ values (Table 1).

**Table 1:** Guinier radii, maximal dimensions, molecular mass estimates derived from SAXS data and molecular masses calculated from amino acid sequences.

	p44	p44 + Rho-PP	R175E	R175E + Rho-PP
*R*_G_ from Guinier analysis (nm)	3.13	3.65	3.20	4.01
*R*_G_ real space from P(r) (nm)	2.85	3.58	3.05	3.75
*R*_G_ rec. space from P(r) (nm)	2.85	3.58	3.05	3.75
*D*_max_ (nm)	8.06	10.42	9.23	10.52
*M*_m_ from Porod volume (kDa)	34.2	70.0	47.9	76.2
*M*_m_ from Bayesian interference (kDa)	41.1	74.3	42.9	85.7
*M*_m_ from ab initio (kDa)	51.7	80.0	49.5	101.8
*M*_m_ from protein sequence (kDa)	41.1*	82.2^#^	47.1*	94.2^#^

* Monomer, ^#^ dimer.

Based on the knowledge that peptide-binding induces arrestin dimerization, we used the available crystal structures of p44 (PDB ID: 3UGU) and R175E (PDB ID: 4ZRG) in dimeric conformations to calculate theoretical SAXS curves that can be compared to the respective measured SAXS data (Fig. 2A,B, Table 2, Supplementary Fig S2). No high resolution structure information is available on R175E mutant or p44 in the presence of rhodopsin phosphopeptide that we could use as dimeric rigid body models for fitting the experimental SAXS data. We therefore considered the possible dimer arrangements observed in closely related proteins. For example, crystal structure of the bovine visual arr-1 (PDB ID 3UGX), which is our wild type protein in which the point mutation R175E was induced by site-directed mutagenesis^19^. For p44, two crystal structures of bovine rod protein are available, in monomeric (PDB ID 3UGU) and in dimeric (PDB ID 4J2Q) form. We therefore selected four dimer conformations available in 3UGX and 4J2Q crystal structures, which are named in the manuscript as follows: ‘N–C’ and ‘C–C’ dimers as in the wild-type arr-1 (PDB ID 3UGX); and ‘C–C’ and ‘N–N’ dimers as in activated p44 (PDB ID 4J2Q). For the fitting of the experimental SAXS data and for the ab initio modelling described below, the dimer conformations based on 3UGU and 4ZRG were generated for p44 and R175E, respectively.

**Table 2:** Normalized spatial discrepancy (NSD) and Chi (χ) values providing goodness of the fits.

Sample	Dimer arrangement	DAMMIF	
		NSD	χ*
P44 + Rho-PP	N–C dimer (as in 3UGX) C–C dimer (as in 3UGX) C–C dimer (as in 4J2Q) N–N dimer (as in 4J2Q)	2.07 2.06 1.98 2.19	0.79 2.51 0.83 0.87
R175E + Rho-PP	N–C dimer (as in 3UGX) C–C dimer (as in 3UGX) C–C dimer (as in 4J2Q) N–N dimer (as in 4J2Q)	3.01 2.54 2.80 3.06	1.31 1.54 1.26 1.29

* χ values given for R175E models do not include the flexible tail region as the EOM calculations generate χ-values of around 0.67 for all models that does not allow differentiation between goodness of fits.

**Figure 3:**
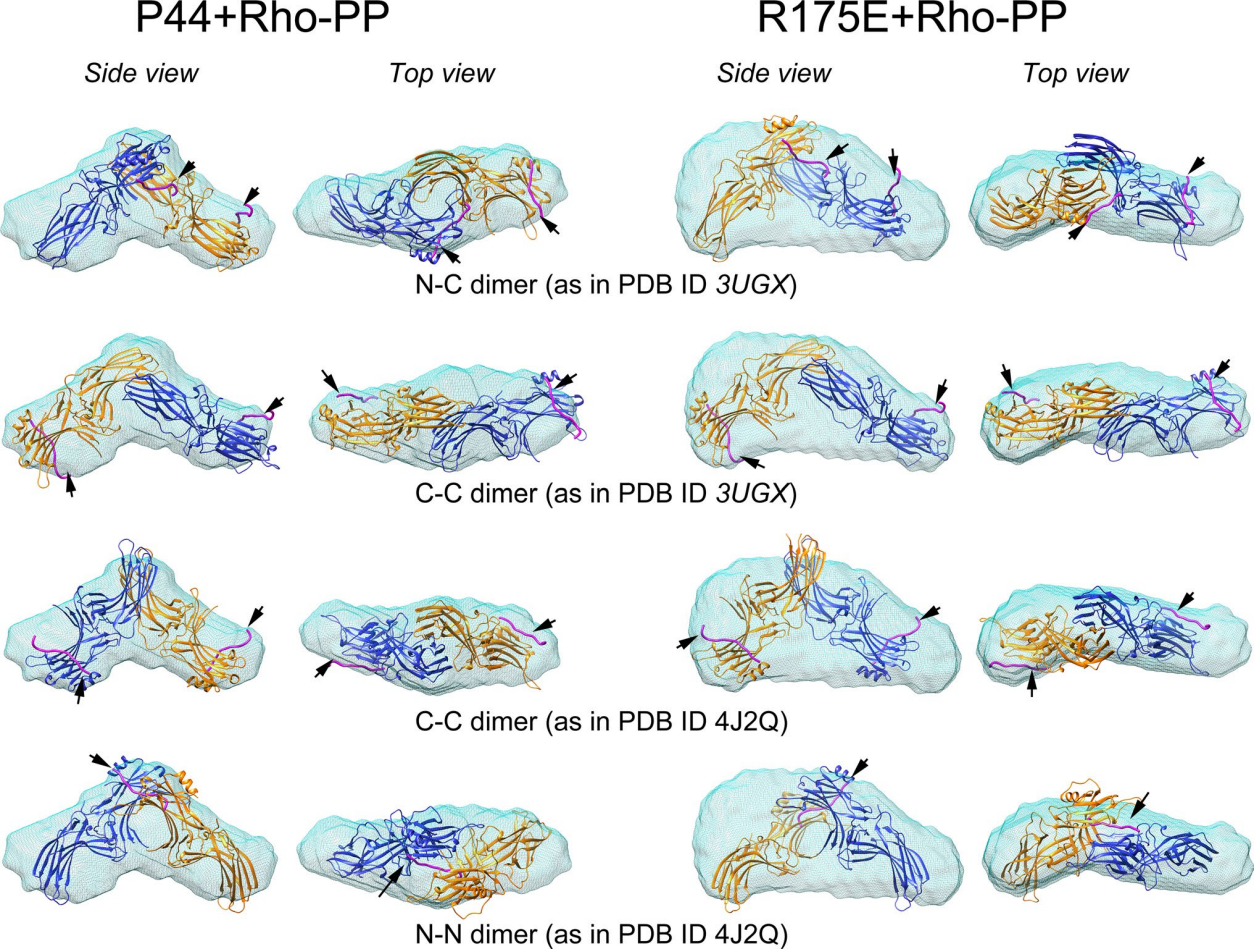
Ab initio envelope reconstructions of p44 and R175E determined by SAXS. Ribbon structures of the respective crystal structures (PDB ID 3UGU for p44 and PDB ID 4ZRG for R175E) are fitted as dimers in the envelope (blue mesh). The figure shows both, side and top views. All p44 envelopes were fitted with p44 crystal structure (PDB ID 3UGU), and R175E envelopes were fitted with R175E crystal structure (4ZRG), in dimeric arrangements as follows (top to bottom): ‘N–C’ dimer as in PDB ID 3UGX (arrestin-1), ‘C–C’ dimer as in PDB ID 3UGX, ‘C–C’ dimer as in PDB ID 4J2Q (p44) and ‘N–N’ dimer as in PDB ID 4J2Q. When bound to two phosphopeptides in 1:1 ratio, the ‘N–N’ dimer shows steric clashes (see Supplementary Fig S3), we thus show here the ‘N–N’ dimer bound to a single phosphopeptide. The two protomers are shown in orange and blue. Approximate location of the rhodopsin phosphopeptide colored magenta (indicated by arrowheads) is derived from superposition of the crystal structure of rhodopsin-arrestin complex PDB ID 5W0P on the respective structures^9^.

NSD values provide a quantitative estimate of the structural agreement between high resolution atomistic and low resolution ab initio models shown in Fig. 3. Fitting accuracy of the high resolution rigid body model with the experimental data shown in Fig. 2 is expressed as χ values.

For the p44 dimer no flexible tails of the protein need to be considered and we calculated the theoretical SAXS diffraction pattern of the truncated protein using the CRYSOL program. For R175E, the flexible C-tails of the full-length protein need to be included into the calculations, and this was done by using the EOM algorithm. The agreement of theoretical SAXS curves with the measured SAXS data is clearly visible in the flat random profile of the residuals (both panels at the bottom of Fig. 2A, B) and the obtained small χ-values (χ = 0.8 for p44 + Rho-PP and χ = 0.67 for R175E + Rho-PP) that indicate an excellent fit of the dimer models to the SAXS data (Table 2). These calculations strongly corroborate and clearly support our observation that peptide-interaction results in dimer formation in both: p44 + Rho-PP and R175E + Rho-PP.

Next, we performed ab initio modelling to visualize the solution structures in real space. The DAMMIF program was used to calculate representative bead models of p44 + Rho-PP and R175E + Rho-PP. Those ab initio models are shown as envelope reconstructions in Fig. 3.

Crystal structures of p44 used in this study has been reported previously (PDB ID 3UGU)^21^ with one molecule per asymmetric unit. We aligned above-mentioned four different dimeric conformations of the p44 structure (PDB ID 3UGU) in the envelope. As shown in Fig. 3, all of the p44 dimeric arrangements align well in the ab initio reconstructions, again supporting presence of a dimer when bound to rhodopsin phosphopeptide. This verifies our observation that the dimeric p44 model provides an excellent fit to the experimental SAXS data in reciprocal space as shown in Fig. 2 (Fig. 2A). We calculated the NSD (normalized spatial discrepancy) values for each fit that provides a quantitative estimate of similarity between the high-resolution atomistic and low-resolution ab initio models (Table 2). Low NSD values indicate that both, the envelope and the fitted dimer show a good structural agreement. The ab initio modelling approach, however, does not possess the inherent structural resolution to allow us to differentiate which particular dimeric orientation fits the best in p44 + Rho-PP envelope, also reflected in the NSD values that show only minor differences (Table 2).

In our previously reported low resolution SAXS envelope of R175E mutant, the protein is a monomer in solution^19^. Alignment of the crystal structure reported in the same study with one molecule per asymmetric unit showed extended space that could not be assigned to residues from the crystal structure. Notably, forty three C-terminal residues including the C-tail (361–404) could not be traced in the electron density maps and thus are likely to be disordered in the crystal structure. The SAXS data of the R175E monomer fitted best when the C-tail was considered flexible (χ = 0.67 for the structure with flexible C-tail vs. χ = 1.70 for the crystal structure only); providing possible explanation of the extended space in the R175E envelope obtained previously by SAXS^19^. Using R175E crystal structure (PDB ID 4ZRG), we aligned the four dimer conformations mentioned above in the ab initio envelope of R175E + Rho-PP (Fig. 3). Both, Fig. 3 and the NSD values given in Table 2 show that resulting alignments are not ideal fits indicating significant differences in the crystal structure and the solution structures. Nonetheless, these results confirm formation of a dimer in the presence of the rhodopsin phosphopeptide as neither a monomer (see Supplementary Fig S4) nor a higher oligomer could be fitted in the envelope. Structural modelling performed in reciprocal space strongly support that R175E forms a dimer and demonstrate that the flexible C-tails need to be included in the modelling calculations (Fig. 2B). Hence, it is tempting to assume that the bulkier shape of the ab initio model as compared to the crystallographic dimers can be attributed to the flexible C-tails. The disordered C-tails lead to the extended and bulkier shape of the ab initio model, which was previously reported in the low resolution SAXS envelope of R175E monomer as well^19^.

**Figure 4:**
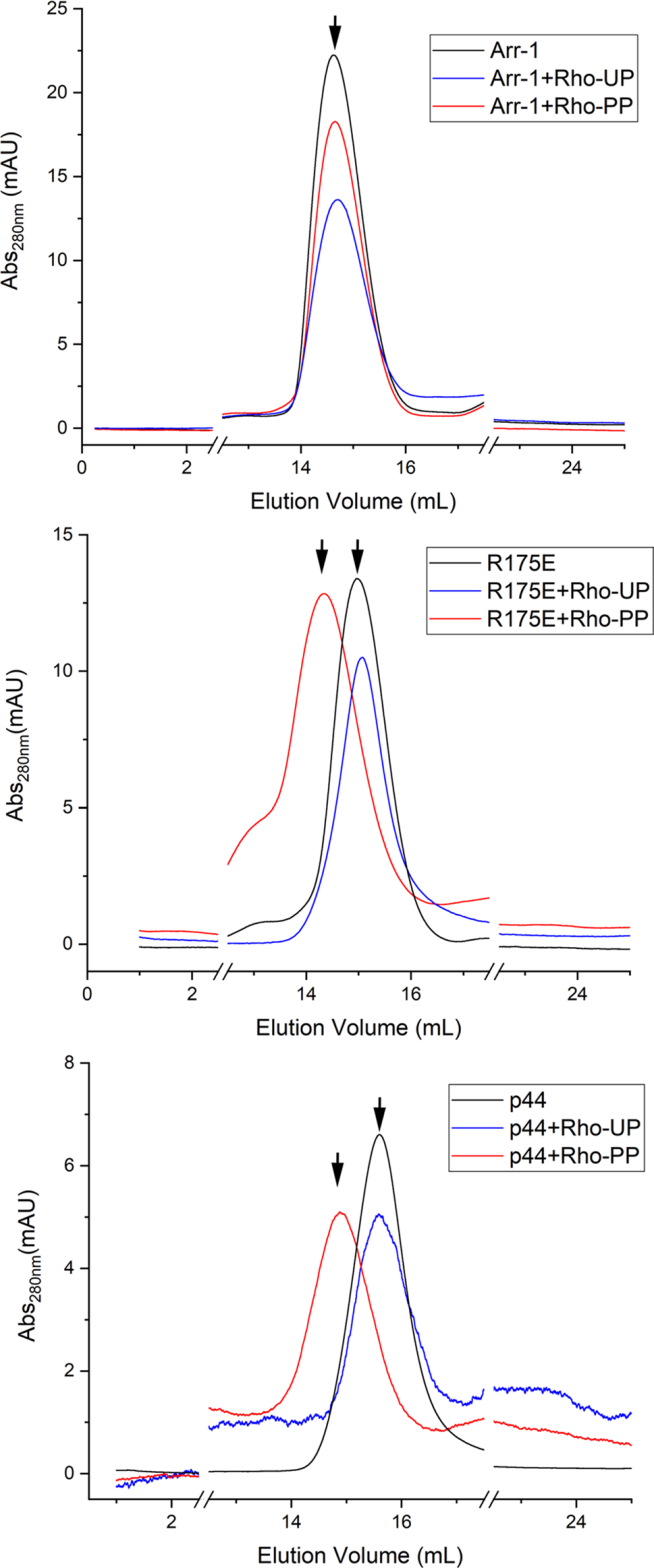
Elution profiles of p44, R175E and arr-1 in the presence and absence of rhodopsin phosphopeptide. Note the shift in elution volumes for p44 and R175E in the presence of phosphorylated peptide Rho-PP. Presence of unphosphorylated peptide (Rho-UP) causes no alteration in the elution profiles for all three proteins.

Size exclusion chromatography was applied to all arrestin proteins (R175E, p44 and wild type arr-1 used as reference), in the presence and absence of phosphopeptide as described in the methods section. Purified fractions were concentrated and applied to a Superdex 200 16/600 column to separate any higher oligomer or aggregate present prior to SAXS measurements. For the wild‐type arrestin, dimer species was detected irrespective of the presence of phosphopeptide. In contrast, both the active arrestin variants show a significant shift in the retention volume in the presence of phosphopeptide (Fig. 4, Supplementary Table S1). In the absence of peptide, p44 and R175E arrestins show the retention volume of 15.5 mL and 15.0 mL, respectively. This corresponds to the approximate molecular weight of ~ 40 and ~ 48 kDa, as expected for the respective monomeric states. The binding of the peptide leads to a shift of the retention volumes to 14.8 mL and 14.3 mL for p44 and R175E, respectively. In contrast, the retention volume of wild type arr-1 remained 14.7 mL in both the conditions that corresponds to an apparent molecular weight of 60 kDa. A mismatch of the apparent molecular weight calculated for arrestin dimer from SEC with the theoretical values in Table 1 is presumably due to the unusual shape and net charge of arrestin molecule, which in turn can have an effect on protein mobility during size exclusion chromatography. We used the standard globular proteins for these calculations. Nevertheless, a significant shift in retention volumes (Fig. 4, Supplementary Table S1) as well as shift in gel mobility in the native-gel electrophoresis (Supplementary Fig S5) clearly indicates the formation of higher species in p44 and R175E in the presence of a rhodopsin phosphopeptide. Even though for the SEC method we cannot exclude presence of an equilibrium between monomer and higher oligomer such as dimer, results mentioned above from SAXS suggest presence of dimers only, with no indications of monomer–dimer equilibrium. Size exclusion chromatography on all three arrestin proteins were also performed in the presence of unphosphorylated peptide which resulted in no change in the retention volumes and were similar to the control runs performed without any peptide (Supplementary Table S1). These results are consistent with the reports that binding of arrestin to the receptor requires phosphorylation of the C-terminus of the receptor^22,23^.

## Discussion

It is highly intriguing how just four members of the arrestin family interact with hundreds of different GPCRs and other signaling proteins to regulate a multitude of physiological processes. Acting as the classical signaling scaffolds, they display high structural conservation with similar overall fold consisting of N- and C- terminal β-sheet domains with a centrally located polar core region (Fig. 1). High sequence and structure homology in arrestin members suggest similar activation mechanism by different GPCRs. Using rhodopsin and visual arr-1 as prototype models for GPCR-arrestin interaction, we have investigated the effect of rhodopsin C-terminal peptide on global conformation of constitutively active variants of arr-1. Using SAXS, we demonstrate that the phosphopeptide induces dimerization in both the pre-active arrestins.

We previously reported crystal structures of bovine p44 splice variant of visual arrestin^21^, and of R175E polar core mutant^19^ where both the preactive variants crystallized as monomers with single molecule per asymmetric unit. Additionally, using SAXS and SEC techniques, we demonstrated that both the proteins are monomers in solution. In contrast, the wild type arr-1 remains a dimer, as also observed in a previous SAXS study^24^. Crystal structures of the respective proteins align well in the ab initio envelopes derived from SAXS^19^. Notably, both p44 and R175E are constitutively active arrestins that can terminate phototransduction by binding to unphosphorylated, light-activated rhodopsin. Crystal structures of both these arrestins show a disrupted polar core and a break in three element interactions^25,26^. The current activation model based on recent structure of the arrestin-rhodopsin complex suggests binding of monomeric arrestin to the activated rhodopsin^27^. Similar 1:1 stoichiometry was also observed for the β2 adrenergic receptor (β2AR) – β-arrestin-1 and, neurotensin receptor 1 (NTSR1) – β-arrestin-2 complexes, resolved with electron microscopy^7,28,29^. In contrast, our SAXS results reveal dimerization in the pre-activated arrestins p44 and R175E in the presence of C-terminus phosphopeptide of rhodopsin. These findings are not in line with the hypothesis where dimers are the inert storage forms that dissociate to form monomers upon receptor activation, which then bind the receptor in 1:1 stoichiometry. The high resolution structures of the arrestin-receptor complexes reported previously are of the engineered complexes composing N-terminally T4 lysozyme-tagged constitutively active variant of GPCR fused to the active variant of arrestin in the C-terminus (T4L-GPCR-arrestin)^7,27^. In our work, we have used active variants of arrestin in the presence of phosphorylated and unphosphorylated rhodopsin peptides. Conceptually, as the C-terminal phosphopeptide residues are present in our work as well as in the receptor in the arrestin-GPCR complexes, one can expect dimerization to occur in both the cases. However, this was not the case in the complexes which support 1:1 stoichiometry. Major difference in these studies lies in the design of the construct where the C-terminus of the GPCR being fused to an arrestin molecule in the T4L-GPCR-arrestin complex may not be available for interactions required for dimerization. Presence of free receptor and arrestin molecules are critical for dimerization to occur that is unlikely for the engineered complexes. Nonetheless, solution NMR spectroscopy studies of arr-1 interaction with phosphorylated rhodopsin were proposed to significantly affect the monomer–dimer-tetramer equilibrium of arr-1 in the photoreceptors^30^. Also, the addition of synthetic receptor phosphopeptides has been shown to modulate arrestin affinity, activation and global conformation^18,31^.

The next logical question that arises here is the possible biological role of receptor-induced arrestin dimers/oligomers, which might be considerably different in visual and nonvisual arrestins. Most of the studies reporting arrestin oligomerization are in vitro studies where self-association has been investigated at relatively high concentrations^20^. For example, arr-1 was crystallized as a tetramer (dimer of dimers) and was reported to form dimers and tetramers in solution^2,3,24,32^, and β-arrestins self-associate and form heterodimers^33^.

Dimers of visual arrestin have been proposed as inert storage forms that can release monomers after dissociation when activated to bind rhodopsin^20^. Self-association of arr-1 was suggested as a cytoprotective mechanism to reduce the concentration of toxic monomers in photoreceptor cells. Although the exact mechanism of monomer toxicity remains to be elucidated, oligomerization of arr-1 prevents harmful interactions^34^. In another hypothesis, dimerization of arrestin provides a platform for the binding of receptor dimers or other downstream effector proteins. Boularan et al. reported specific interaction of Mdm2 with β-arrestin-2 oligomers that leads to the formation of higher-order functional complexes^35^. Introduction of mutations that impair β-arrestin oligomerization result in reduced interaction with Mdm2, and inhibit p53-dependent antiproliferative effects of β-arrestin in tumor cells. Intracellular concentration of β-arrestin-2 oligomers was thus proposed to control cell survival and proliferation.

Fotiadis et al. reported dimerization of the GPCR rhodopsin in the rod outer segment (ROS) disk membranes using AFM and cryo-electron tomography techniques^36,37^. Cryo-EM structures of a rhodopsin dimer reconstituted into nanodiscs from purified monomers and of the cross-linked rhodopsin dimer were reported recently where the primary intradimeric interface is formed by TM1and H8 of each rhodopsin molecule^38^. Overlaying crystal structure of the arrestin-rhodopsin complex on rhodopsin dimer, the authors propose that two arrestin molecules may bind a receptor dimer, where the N- β-sheet domains form the interface. Such an N–N mediated arrestin dimer, however, has an extended conformation that does not fit in our SAXS envelope. The other N–N arrestin dimer of 4J2Q shows steric clashes between the bound phosphopeptides (Supplementary Figure S3). It is thus unlikely that an arrestin dimer in this arrangement can bind two receptors. However, it remains possible that two arrestin molecules bind a single phosphopeptide as shown in bottom panel of Fig. 3. The N–C dimer and the two C–C dimer conformations are accommodated equally well in the ab initio envelopes, and show similar NSD values (Table 2). Based on the crystal structure of arrestin-rhodopsin complex, binding of the phosphopeptides to the N–C dimer is antiparallel. When bound, one of the arrestin molecules thus will be rotated away from the disc membrane. Such a scenario is unlikely. The C–C dimer of 3UGX fits poorly to the experimental SAXS data, resulting in high χ values for both, P44 and R175E (Table 2 and Supplementary Fig S2); and do not support existence of such a dimer either. In contrast, the C–C dimer of 4J2Q exhibits very good agreement with both, the experimental data and the ab initio envelopes (Fig. 2 and Table 2). The distance between the two phosphopeptides (measured from the center) in this dimer is ~ 88 Å. This is too long as the approximate distance between two rhodopsin molecules in a dimer is ~ 38 Å. For C–C arrestin dimer it is theoretically possible that the two arrestin molecules bind the rhodopsin molecules from adjacent dimers. Interestingly, the p44 crystals that resulted in the C–C dimeric arrangement in 4J2Q were obtained in the presence of opsin in the crystallization condition^26^. Though the crystals contained only p44 and no opsin, presence of latter in the crystallization setup might have induced the crystal packing that allows dimer formation. The crystal structure obtained reveals pre-activated conformation with ~ 20° inter-domain rotation. The C–C dimers are in agreement with SAXS investigations performed by Shilton et al. on visual arr-1^24^. The results on wild type arr-1 also agree with our observations where addition of phosphopeptide involves only small and localized conformational changes with no changes in the oligomeric state. Given the high plasticity in arrestin structures, it is conceivable that structural changes within the dimers such as intra and interdomain rotations allow interaction of arrestin dimers with two rhodopsin molecules arranged in the rows as observed in the native disc membranes^37^. The binding stoichiometry between GPCRs and arrestins still remains to be fully established. Dimerization might play a role in the allosteric recruitment of arrestin molecules upon phototransduction. Other cellular functions such as evident in case of β-arrestins might involve receptor internalization, and recruitment of downstream effector molecules.

## Methods

Expression and purification of wild type, R175E mutant and p44 splice variant: Recombinant arr-1, mutant R175E and p44 were cloned into the pYEX-BX vector (Clontech, Heidelberg), as described previously^19^. Large-scale expression and purification of all recombinant arrestins was performed in *Saccharomyces cerevisiae* F11 α strain as described before^39^. To avoid any higher oligomers and aggregate formation, only freshly purified proteins were used. Purified arrestin preparations were subjected to size exclusion chromatography (SEC) on a Superdex 200 16/600 column (GE, Healthcare Life Sciences) using 10 mM Hepes, 150 mM KCl, pH 7.5 buffer on an AKTA purifier chromatography system (GE, Healthcare Life Sciences). SEC was performed in both, the presence and in the absence of the phosphopeptide. A two-fold molar excess of phosphopeptide was added to purified arrestins, and mixture was incubated for 2–3 h at room temperature prior to SEC. The flow rate throughout was 0.5 ml min^−1^, and 0.5 mL fractions were collected. Absorbance was monitored at 280 nm and the presence of protein in peak fractions was verified by 12% SDS-PAGE. Subsequently, selected protein fractions were concentrated to the desired protein concentration using Amicon-4-cut off 10 kDa (Millipore) ultrafiltration devices as per manufacturer’s protocol.

The synthetic peptides corresponding to C-terminal residues 323–348 from bovine rhodopsin were purchased from CASLO ApS, Denmark. The sequences of unphosphorylated and phosphorylated peptides are CGKNPLGDDEASTTVSKTETSQVAPA and CGKNPLGDDEA(Sp)(Tp)(Tp)V(Sp)K(Tp)E(Tp)(Sp)QVAPA, with acetylated N-terminus in both the peptides.

The synchrotron SAXS data was collected at beamlines BM29 at the ESRF (Grenoble, France)^40^ and P12 operated by EMBL Hamburg at the PETRA III storage ring (DESY, Hamburg, Germany)^41^ using protein from two different protein purifications. The X-ray wavelengths used on P12 and BM29 were 1.24 Å and 0.992 Å, respectively. Sample to detector distance on P12 was 3 m, and on BM29 it was 2.872 m. Temperature in the sample holder was kept at 20 °C throughout all experiments. Ten individual frames with 3 s exposure time were recorded on BM29, while on P12 twenty frames with exposure time 0.1 s each were measured. The samples were continuously purged through a quartz capillary to minimize possible radiation damage. The frames without radiation damage were averaged, and contribution of the buffer was subtracted from measured intensities of the protein solution. Data were scaled by the measured protein concentrations and extrapolated to infinite dilution. Different protein concentrations were measured (R175E: 0.6 and 1.2 mg/mL, p44: 0.7 and 1.1 mg/mL; R175E + Rho-PP: 0.58 and 0.93 mg/mL; p44 + Rho-PP: 0.6 and 1.0 mg/mL) in buffer containing 10 mM HEPES, 150 mM KCl, pH 7.5. BM29 SAXS data showed better statistics at large q-values, while the P12 SAXS data extends to smaller q-vectors due to the larger X-ray wavelengths and the longer sample to detector distance. BM29 and P12 SAXS data scaled by the protein concentration were overlapping within the error bars. Both the data sets were combined to expand the *q*-range and to minimize the statistical errors.

SAXS data were analyzed using the programs available within the ATSAS software package^42^. Scattering curves of the crystal structure of p44 were calculated and fitted to the experimental SAXS data using the computer program CRYSOL. The distance distribution function P(r) and the Porod volume of the protein were determined with the programs GNOM and DATPOROD, respectively. Ab initio models were generated using the DAMMIF program where twenty models were generated, averaged and the filtered model was used. In brief, a large number of beads with radii of a few Angstrom were allowed to be arranged around by a Monte Carlo algorithm and the obtained structural models were evaluated against the experimental SAXS data as quality criterion, while maintaining compactness of the structure. Twenty models obtained in this approach were then averaged to yield the final averaged ab initio bead model with a uniform bead radius of 3.5 Å. The envelope function of the filtered ab initio model was visualized using the SITUS package^43^. The molecular mass was estimated from the excluded volume of the filtered ab initio model and from the Porod volume using division factors of 2 and 1.7, respectively^42^. Flexibility of the C-tail of R175E arrestin was modelled using the program EOM as described previously^19^. The phosphopeptide secondary structure and binding site was essentially derived from the corresponding region of the arrestin-rhodopsin complex^9^(PDB ID: 5W0P), where residues 330–343 of the C-terminus phosphopeptide of rhodopsin form an extended intermolecular β sheet with the N-terminal β strands of arrestin. The seven N-terminal and five C-terminal residues of the rhodopsin phosphopeptide were not observed in the complex structure and are likely disordered. We modelled these residues with the program EOM as described previously for the C-tail residues of R175 arrestin^19^.

Atomistic dimer crystal structures were aligned into the ab initio bead models using the computer program SUPCOMB^44^ and the normalized spatial discrepancy (NSD) was calculated. The NSD value is a quantitative measure allowing an estimate of the structural agreement between high-resolution atomistic and low-resolution bead models. A low value for the NSD (∼1) indicates that the models are similar to each other. Figures were generated with UCSF Chimera^45^ using secondary structure assignments given by the DSSP program^46^.

## Supplementary information

Supplementary information.

## Abbreviations

arr-1: Visual arrestin-1
GPCR: G-protein-coupled receptor
p44: Splice variant of visual arrestin
Rho-PP: Phosphorylated peptide derived from C-terminus of rhodopsin
SAXS: Small angle X-ray scattering
SEC: Size exclusion chromatography
