# Supplementary material for: Phosphorylated peptide of G protein-coupled receptor induces dimerization in activated arrestin
Source: Sci Rep. 2020 Jul 2;10:10938. doi: 10.1038/s41598-020-67944-0 (PMC7331637; doi:10.1038/s41598-020-67944-0)
Supplement: Supplementary file 1 — Supplementary information. [file 41598_2020_67944_MOESM1_ESM.docx]

**Phosphorylated peptide of G protein-coupled receptor induces dimerization in activated arrestin**

Andreas M. Stadler^1,2^, Joachim Granzin^3^, Anneliese Cousin^3^, Renu Batra-Safferling^3*^

^1^Jülich Centre for Neutron Science (JCNS-1) and Institute of Complex Systems (ICS-1), Forschungszentrum Jülich, D-52425 Jülich, Germany

^2^Institute of Physical Chemistry, RWTH Aachen University, Landoltweg 2, 52056 Aachen, Germany

^3^Institute of Biological Information Processing, IBI-7: Structural Biochemistry, Forschungszentrum Jülich, D-52425 Jülich, Germany

* Corresponding author

Phone: +49-2461-61-9495 Email: [r.batra-safferling@fz-juelich.de](mailto:r.batra-safferling@fz-juelich.de)

SHORT TITLE: GPCR phosphopeptide induces dimerization in arrestin

KEYWORDS: arrestin, dimerization, GPCR, p44, rhodopsin, SAXS, splice variant

**Supplementary Information**

Figure S1. SAXS data of (A) p44 monomer and (B) R175E without peptide.


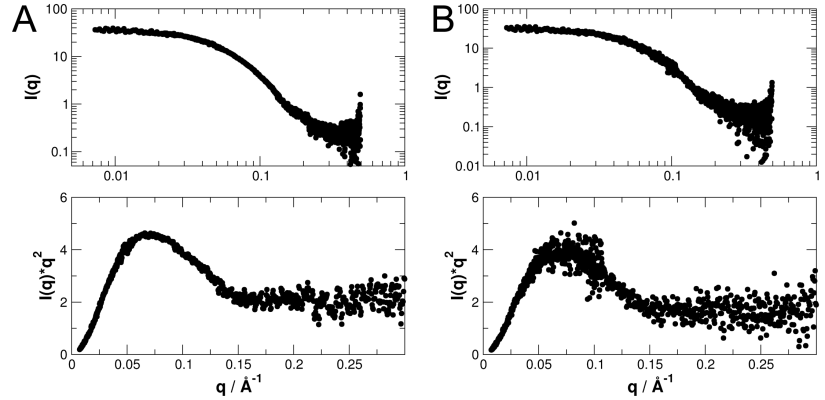


Figure S2. Small-angle scattering data of peptide bound arrestins: p44 and R175E.


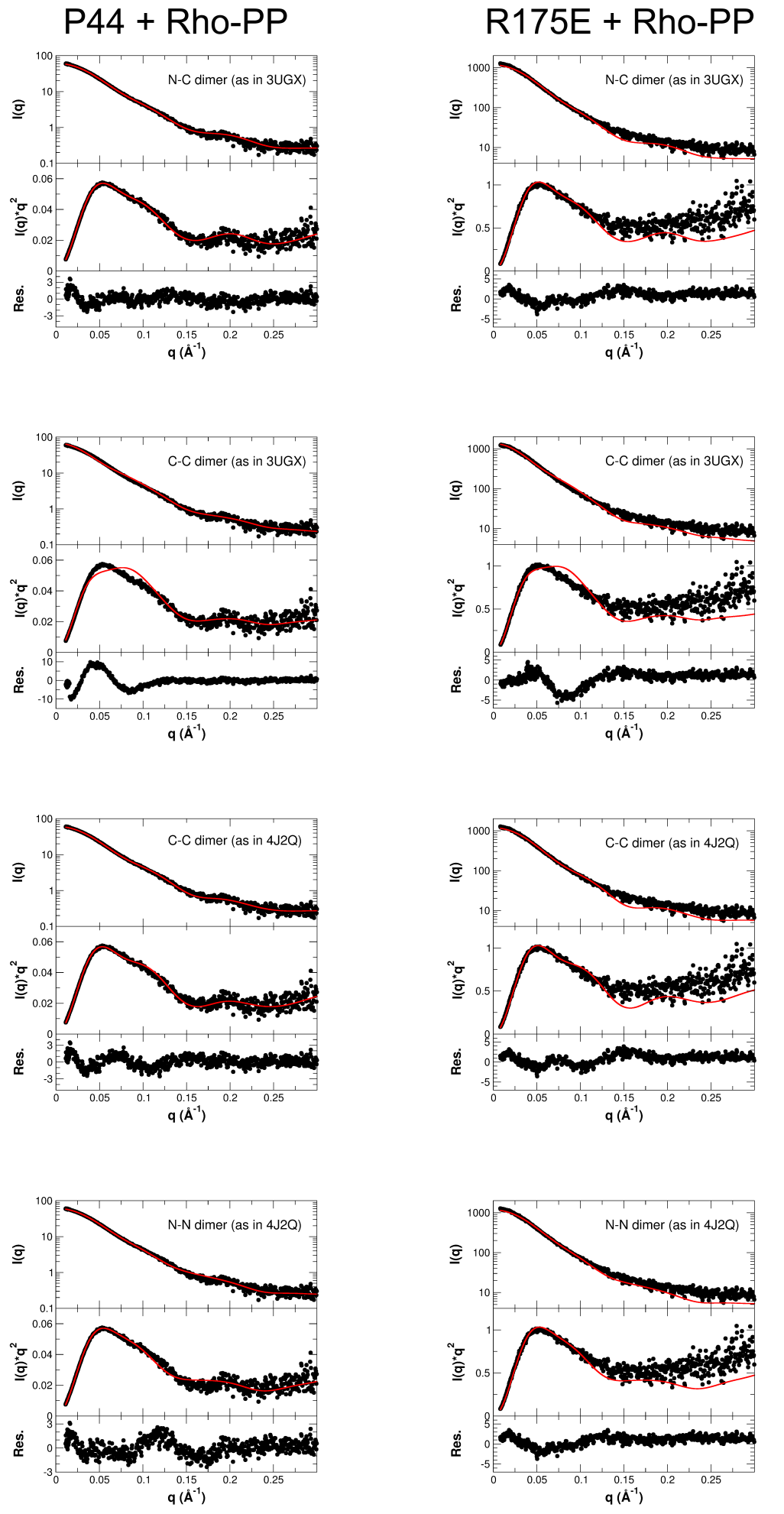


Experimental data and theoretical scattering curves for four different dimer arrangements (solid lines) of p44+Rho-PP (PDB ID 3UGU), and R175E+Rho-PP (PDB ID 4ZRG) are shown. R175E structure does not include the flexible C-tail residues*. The upper panels visualize the measured data on a logarithmic scale, while Kratky plots are shown in the middle panels. The lower panels display the residuals to illustrate the goodness of the structure-based fits to the experimental data.

The crystal structure based dimeric arrangements are shown as ribbon representations in Fig 3.

* The flexible regions of the R175E + Rho-PP SAXS data are not considered in the theoretical dimer models. This results in larger χ values as given in Table 2. Modelling the flexible tails by using the EOM software yields χ-values of around 0.67 for all R175E dimer models, which does not allow differentiation between goodness of fit.

Figure S3. Ab initio envelope reconstructions of P44 and R175E determined by SAXS: the N-N dimer of 4J2Q shows steric clashes.


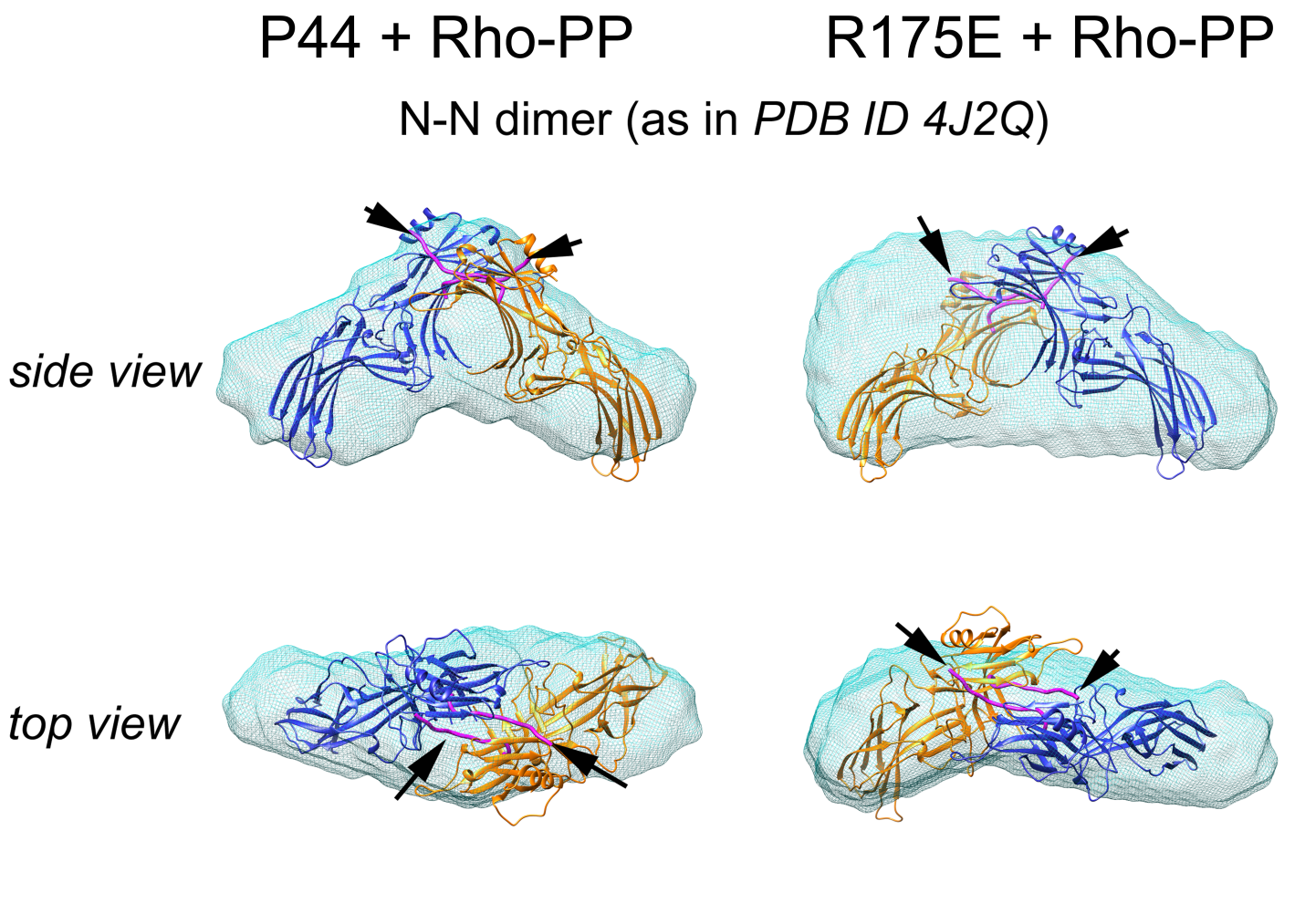


Ribbon structures of the respective crystal structures (PDB ID 3UGU for p44 and PDB ID 4ZRG for R175E) are fitted as dimers in the envelope (blue mesh). The figure shows both, side and top views. The dimeric arrangement used here is the ‘N-N’ dimer as in PDB ID 4J2Q. The two protomers are shown in orange and blue. Approximate location of the rhodopsin phosphopeptide colored magenta (indicated by arrows) is derived from superposition of the crystal structure of rhodopsin-arrestin complex PDB ID 5W0P on the respective structures.

The phosphosphopeptides when bound to arrestin at the ratio of 1:1 as above shows steric clashes in the N-N dimer of 4J2Q. It is thus unlikely that an arrestin dimer in this arrangement can bind two receptor peptides simultaneously.

Figure S4. SAXS data of dimeric (A) p44 + Rho-PP and (B) R175E + Rho-PP. Solid red lines are theoretical curves calculated from the p44 monomer using CRYSOL and of the R175E monomer including flexible C-tail using EOM.


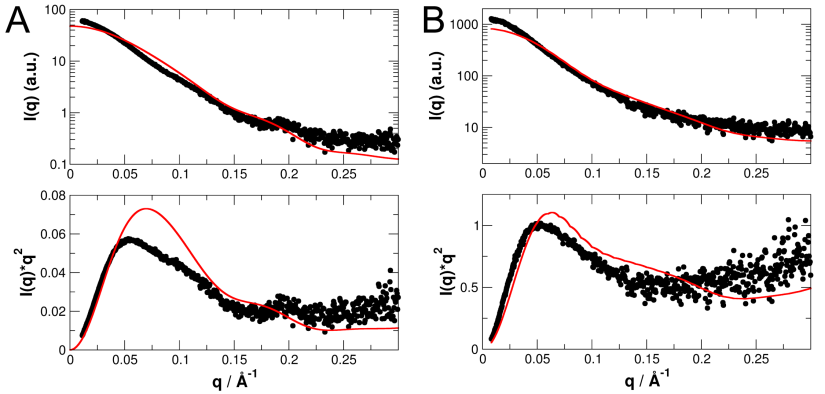


Figure S5. Native gel electrophoretic mobility shift of p44 and R175E arrestin in the presence of rhodopsin phosphopeptide.


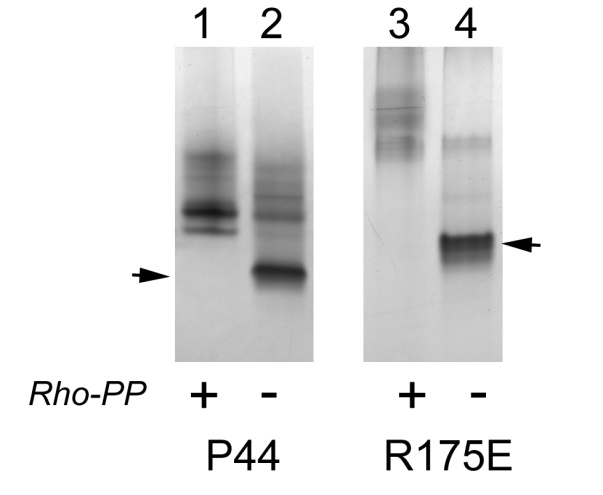


Purified arrestin (10 μM) proteins in the presence or absence of 20 μM Rho-PP were loaded on 4-20% Tris-Glycine gradient gels (Invitrogen) and electrophoresis was performed according to manufacturer’s protocol. The run was performed in cold room at 125 volts for about 135 minutes.

The gel above shows Rho-PP dependent shift in gel mobility for both, p44 and R175E.These observations are consistent with the SAXS and SEC results.

Table S1: Size exclusion chromatography elution volume (Ve) and apparent MW for arr-1, p44 and R175E in the presence and absence of rhodopsin C-terminus peptide.

| Size exclusion chromatography | | | | | | |
| --- | --- | --- | --- | --- | --- | --- |
|  | Without peptide | | + unphosphorylated peptide  (Rho-UP) | | + phosphorylated peptide  (Rho-PP) | |
|  | Elution volume (mL) | app M_w_ (kDa) | Elution volume (mL) | app Mw (kDa) | Elution volume (mL) | app Mw (kDa) |
| Arr-1 | 14.67 | 60 | 14.71 | 60 | 14.66 | 60 |
| R175E | 14.98 | 48 | 14.95 | 49 | 14.3 | 72 |
| p44 | 15.57 | 40 | 15.53 | 40 | 14.8 | 55 |
